# Atypical hemolytic uremic syndrome in children: complement mutations and clinical characteristics

**DOI:** 10.1007/s00467-012-2131-y

**Published:** 2012-03-13

**Authors:** Lianne M. Geerdink, Dineke Westra, Joanna A. E. van Wijk, Eiske M. Dorresteijn, Marc R. Lilien, Jean-Claude Davin, Martin Kömhoff, Koen Van Hoeck, Amerins van der Vlugt, Lambertus P. van den Heuvel, Nicole C. A. J. van de Kar

**Affiliations:** 1 Department of Pediatric Nephrology, Radboud University Nijmegen Medical Centre, PO Box 9101, 6500 HB Nijmegen, The Netherlands; 2 Department of Pediatric Nephrology, VU Medical Centre, Amsterdam, The Netherlands; 3 Department of Pediatric Nephrology, Sophia Children’s Hospital, University Medical Centre Erasmus MC, Rotterdam, The Netherlands; 4 Department of Pediatric Nephrology, Wilhelmina Children’s Hospital, University Medical Centre Utrecht, Utrecht, The Netherlands; 5 Department of Pediatric Nephrology, Emma Children’s Hospital-Academic Medical Centre, University of Amsterdam, Amsterdam, The Netherlands; 6 Department of Pediatric Nephrology, Queen Fabiola Academic Children’s Hospital, Free University of Brussels, Brussels, Belgium; 7 Department of Pediatric Nephrology, University Medical Centre Groningen, Groningen, The Netherlands; 8 Department of Pediatric Nephrology, University Hospital Antwerp, University of Antwerp, Antwerp, Belgium; 9 Department of Pediatrics, Antonius Hospital, Sneek, The Netherlands; 10 Department of Pediatrics, University Hospital Leuven, Leuven, Belgium

**Keywords:** Atypical HUS, Complement regulation, Plasma therapy, Dialysis, Transplantation, Clinical outcome

## Abstract

**Background:**

Mutations in complement factor H (*CFH*), factor I (*CFI*), factor B (*CFB*), thrombomodulin (*THBD*), *C3* and membrane cofactor protein (*MCP*), and autoantibodies against factor H (αFH) with or without a homozygous deletion in CFH-related protein 1 and 3 (*∆CFHR1/3*) predispose development of atypical hemolytic uremic syndrome (aHUS).

**Methods:**

Different mutations in genes encoding complement proteins in 45 pediatric aHUS patients were retrospectively linked with clinical features, treatment, and outcome.

**Results:**

In 47% of the study participants, potentially pathogenic genetic anomalies were found (5x*CFH*, 4x*MCP,* and 4x*C3*, 3x*CFI*, 2x*CFB*, 6xαFH, of which five had *∆CFHR1/3*); four patients carried combined genetic defects or a mutation, together with αFH. In the majority (87%), disease onset was preceeded by a triggering event; in 25% of cases diarrhea was the presenting symptom. More than 50% had normal serum C3 levels at presentation. Relapses were seen in half of the patients, and there was renal graft failure in all except one case following transplant.

**Conclusions:**

Performing adequate DNA analysis is essential for treatment and positive outcome in children with aHUS. The impact of intensive initial therapy and renal replacement therapy, as well as the high risk of recurrence of aHUS in renal transplant, warrants further understanding of the pathogenesis, which will lead to better treatment options.

## Introduction

Atypical hemolytic uremic syndrome (aHUS) is a rare disease characterized by hemolytic anemia, thrombocytopenia, and acute renal failure secondary to thrombotic microangiopathy [1]. It is distinguished from typical or Shigatoxin-producing *Escherichia coli* (STEC) HUS by the absence of STEC infection. In recent years, aHUS has been found to be associated with complement alternative pathway dysregulation. In almost 60% of aHUS patients, mutations in genes encoding complement-regulating proteins are reported [2, 3]. Aside from mutations in these genes, aHUS has also been reported in cobalamin C (cblC) disease caused by mutations in the cblC *MMACHC* gene [4].

With respect to complement-regulating proteins, most mutations are seen in factor H (*CFH*) [3, 5, 6], but mutations in membrane cofactor protein (*MCP* or *CD46*) [3, 5–9] and factor I (*CFI*) [3, 5, 6, 9–11] are also known to predispose development of aHUS. Additionally, gain-of-function mutations in factor B (*CFB*) [6, 12, 13] and complement C3 (*C3*) are reported [6, 14]. Some patients have combined mutations. Functional CFH deficiency due to autoantibodies against CFH (αFH) have recently been described, which is highly associated with a polymorphic homozygous deletion of genes encoding CFH-related protein 1 and 3 (*∆CFHR1/3*) [15–18]. Finally, mutations in the thrombomodulin gene (*THBD*), encoding membrane-bound thrombomodulin that modulates complement activation on cell surfaces, have been reported in aHUS [19]. Genetic complement anomalies are thought to be risk factors rather than the only cause of the disease, as patient relatives with the same mutation do not always exhibit disease activity. AHUS represents approximately 10% of pediatric HUS cases. More than 80% of cases are sporadic [2], with a better prognosis overall than familial cases, which have a higher mutation rate [6]. Knowledge about the impact of mutations in complement-regulating genes upon disease characteristics, response to treatment, and outcome in pediatric aHUS patients could help define therapeutic guidelines and be of prognostic value. Therefore, the aim of this study was to: (1) document the frequency of known genetic complement abnormalities in a cohort of Dutch and Belgian children diagnosed with aHUS, and (2) describe and compare disease characteristics, treatment, and clinical outcome of these children.

## Methods

### Study population

From 2001 to 2010, genomic DNA was analyzed for genetic disorders in complement-regulating genes (*CFH, CFI, MCP, CFB, C3, THBD*, and *∆CFHR1/3* with αFH) in 45 Dutch and Belgian pediatric patients diagnosed with aHUS by pediatric nephrologists at six university medical centers in The Netherlands and one in Belgium. Diagnosis of aHUS was based on the following findings: hemolytic anemia characterized by a hemoglobin (Hb) level <10 g/dl, thrombocytopenia with a platelet count <150 x 10^9^/l, elevated levels of lactate dehydrogenase (LDH), and a negative Coombs test, all in association with acute renal impairment. Patients with STEC HUS were excluded by stool cultures, polymerase chain reaction (PCR) for Shiga toxin, and/or serologic antibodies against LPS O157. Genetic analyses were combined with retrospectively obtained information about patient clinical presentation, received treatment, and outcome. Genetic analysis of 25 of these 45 patients has been published before in a cohort of adults and children by Westra et al. [9]. Patients in that and this study are indicated in the authors’ earlier study [9] by the following numbers 2, 3, 4, 5, 6, 9,15, 16, 18, 21, 24, 33, 36, 39, 40, 41, 42, 43, 44, 45, 46, 48, 54, 58, 62. When two or more members of the same family were affected by the disease at least 6 months apart, it was considered as familial aHUS. Sporadic aHUS patients had no family history of the disease. Complete remission was defined by normalization of hematologic parameters (Hb >10 g/dl, thrombocytes >150x10^9^/l, LDH <450U/l) and renal function [glomerular filtration rate (GFR) >80 ml/min/1.73 m^2^, no proteinuria]. In patients with partial remission, hematologic normalization was seen, but with renal sequelae (GFR <80 ml/min/1.73 m^2^ and/or proteinuria and/or hypertension). The presence of proteinuria was defined as >0.2 g/24 h or positive dipstick for proteins. Hypertension was defined as a consistent blood pressure >p95 for age and length. Recurrence was reported when a new episode of aHUS presented >4 weeks after remission. Chronic treatment was defined by the use of dialysis or plasma therapy for >3 months. The average follow-up time was 7.5 years, varying from two weeks in one patient previously diagnosed and still hospitalized at time of inclusion, to 17 years in one patient who reached adulthood. Permission to study DNA material was given by all patients and/or their parents.

### Genetic analysis of genes encoding *CFH*, *CFI*, *MCP*, *C3*, *CFB*, and *THBD*

Genomic DNA was amplified for *CFH* [National Center for Biotechnology Information (NCBI) RefSeq NM_000186.3], *CFI* (NM_000204.3), *MCP* (NM_002389.3), *C3* (NM_000064.2), *CFB* (NM_001710.4), and *THBD* (NM_000361.2) by means of PCR. Primer data are available upon request. Fragments included DNA sequences of the individual exons and the splice donor and acceptor site. Amplimers were subjected to double-stranded DNA (dsDNA) sequence analysis on an ABI 3130 *xl* Genetic Analyzer (Applied Biosystems). Sequence analysis was performed using Sequencher 4.8 software. Detected genetic aberrations were confirmed on a second PCR product. Genomic DNA from >100 healthy ethnically matched control individuals was used to confirm sequence variations that might be potentially pathogenic. Potential pathogenicity of genetic alterations was further checked in the literature, evolutionary conservation, and *in silico* prediction programs (Sorting Intolerant From Tolerant [SIFT; http://sift.jcvi.org/] and Polymorphism Phenotyping v2 [PolyPhen-2; http://genetics.bwh.harvard.edu/pph2/]). For evolutionary conservation, the 17-way vertebrate alignment from the University of California Santa Cruz (UCSC) Genome Browser (http://genome.ucsc.edu) was used.

### Autoantibodies against factor H (αFH) and homozygous deletion of *CFHR1* and *CFHR3* genes

Serum samples were tested for the presence of αFH by means of enzyme-linked immunosorbent assay (ELISA), as described previously [20]. A positive control sample was kindly obtained via Dr. Dragon-Durey (Paris, France). Test results were considered positive if they were above twice the standard deviation (SD) calculated from controls, and samples were tested at least three times independently, as described by Dragon-Durey et al. [15]. Homozygous deletion of *CFHR1* and *CFHR3* was identified as described previously [9]. In short, genomic DNA was amplified by PCR using specific primers located in a 100-kb region downstream of *CFH*. Amplification of one of the fragments failed in case of Δ*CFHR1/3*. This method does not detect a heterozygous deletion of the two genes or the presence of a hybrid *CFH/CFHR1* gene.

## Results

### Patients

Data on *CFH*, *CFI*, *MCP*, *CFB*, and *C3* mutation characteristics are reported in Table 1. Data on patient characteristics at presentation, treatment, and clinical outcome for each type of mutation are reported in Tables 2, 3, 4. Due to the small number of patients per mutated complement protein, data were analyzed descriptively without statistical comparisons. Clinical data on the four patients with a combined mutation or a mutation in combination with a homozygous deletion in *CFHR1/3* and αFH are given in Table 5. Forty-five patients diagnosed with aHUS from six different university medical centers in The Netherlands and one in Belgium were included. Approximately as many males as females were included in this study.

**Table 1:** Characteristics of mutations found in *CFH*, *CFI*, *MCP*, *CFB,* and *C3* genes in 17 of 45 pediatric patients with atypical hemolytic uremic syndrome (aHUS)

Subject code, exon/intron	Mutation	Effect	Known/unknown
Complement factor H			
Exon 23 (*n* = 1)	c.3628C > T	p.Arg1210Cys	Known
Exon 23 (*n* = 3)	c.3572C > T	p.Ser1191Leu	Known
Exon 9 (*n* = 1)	c.1198C > A	p.Gln400Lys	Known
Complement factor I			
Intron12 (*n* = 1^a^)	g.IVS12 + 5G > T	Splice score decrease from 0.93 to 0.86	Known
Exon 9 (*n* = 1^b^)	c.1019T > C	p.Ile340Thr	Known
Exon 10 (*n* = 1^c^)	c.1071T > G	p.Ile357Met	Unknown
Membrane cofactor protein			
Exon 6 (*n* = 1^a^)	c.811-816delGACAGT	p.delAsp271-Ser272	Known
Complement C3			
*n* = 4^b^	c.481C > T	(p.Arg161Trp)	Known [29]
Complement factor B			
*n* = 2	c.967A > G	p.Lys323Glu	Known

Every single mutation is mentioned separately. Patients without mutations but with a homozygous deletion in *∆CFHR1/3* and/or autoantibodies against factor H are not listed in this table. Of three patients with an *MCP* mutation, DNA analysis was not performed in our laboratory. Mutation and effect are therefore not listed above

^a^Patient carrying both *CFI* and *MCP* mutations

^b^One patient carrying both *CFI* and *C3* mutations

^c^Patient carrying *CFI* mutation and a homozygous deletion in *∆CFHR1/3* and autoantibodies against *CFH* (αFH)

According to www.fh-hus.com and [29]

**Table 2:** Characteristics at onset of atypical hemolytic uremic syndrome (aHUS) in 45 pediatric patients with mutations in *CFH*, *CFI*, *MCP*, *CFB*, and *C3*, with a homozygous deletion in *CFHR1/3* (*∆CFHR1/3*) and autoantibodies against CFH (αFH), as well as in patients without known genetic anomalies

Parameter	All patients (no.)	No genetic defect (no.)	Genetic defect (no.)	*CFH* (no.)	*CFI* (no.)	*MCP* (no.)	*CFB* (no.)	*C3* (no.)	*∆CFHR1/3* (no.)	αFH (no.)
Male/female	22/23 (45)	12/13 (25)	10/10 (20)	2/3 (5)	3/0 (3)	3/1 (4)	0/2 (2)	2/2 (4)	3/2 (5)	3/3 (6)
Familial/sporadic	11/34 (45)	2/23 (25)	9/11 (20)	3/2 (5)	1/ 2 (3)	0/4 (4)	2/0 (2)	4/0 (4)	0/5 (5)	1/5 (6)
Age of onset										
0-1 year	10 (45)	9 (25)	1 (20)	0 (5)	0 (3)	0 (4)	0 (2)	1 (4)	0 (5)	1 (6)
1-7 years	24 (45)	10 (25)	14 (20)	4 (5)	2 (3)	2 (4)	1 (2)	2 (4)	5 (5)	5 (6)
≥7 years	11 (45)	6 (25)	5 (20)	1 (5)	1 (3)	2 (4)	1 (2)	1 (4)	0 (5)	0 (6)
Symptoms										
Fever	13 (41)	6 (22)	7 (20)	0 (4)	1 (3)	1 (4)	0 (2)	4 (4)	2 (5)	3 (6)
URTI	14 (31)	5 (14)	9 (19)	2 (5)	2 (3)	1 (2)	2 (2)	3 (4)	1 (3)	2 (4)
Oligo/anuria	28 (40)	15 (21)	13 (19)	5 (5)	1 (3)	2 (3)	1 (2)	3 (4)	2 (5)	3 (6)
Gastrointestinal^a^	32 (43)	17 (23)	15 (20)	3 (5)	2 (3)	2 (4)	1 (2)	4 (4)	4 (5)	5 (6)
Hypertension	32 (45)	20 (25)	12 (20)	3 (5)	1 (3)	1 (4)	1 (2)	2 (4)	5 (5)	6 (6)
Biochemical evaluation (mean levels)										
Hemoglobin (g/dl)	7,6 (45)	7,7 (25)	7,5 (20)	7,0 (5)	12,4 (3)	13,7 (4)	12,2 (2)	10,6 (4)	12,7 (5)	12,2 (6)
Platelet count (×10^9^/l)	81 (45)	86 (25)	76 (20)	69 (5)	136 (3)	29 (4)	175^b^ (2)	114 (4)	51 (5)	50 (6)
LDH (U/l)	3784 (42)	4382 (23)	3062 (19)	1338 (5)	4639 (3)	3688 (3)	1407 (2)	3195 (3)	5147 (5)	4886 (6)
Uremia (mmol/l)	28 (45)	31 (25)	25,6 (20)	27 (5)	28.5 (3)	19,8 (4)	46,6 (2)	19,4 (4)	35,1 (5)	31,5 (6)
Creatinine (mmol/l)	248 (45)	290 (25)	195 (20)	193 (5)	155 (3)	124 (4)	459 (2)	100 (4)	225 (5)	199 (6)
C3 (mg/l)^c^	881 (37)	862 (22)	908 (15)	1276 (5)	805 (2)	933 (3)	335 (2)	810 (3)	717 (3)	705 (4)
C4 (mg/l)^d^	232 (31)	227 (19)	240 (12)	220 (3)	193 (2)	185 (1)	378 (2)	230 (1)	227 (3)	227 (3)

The numbers of patients for whom data were available are reported in parentheses

*URTI* upper respiratory tract infection, *LDH* lactate dehydrogenase

^a^Vomiting and/or diarrhea

^b^Although one criterion of having aHUS is to have serum platelet count <150 × 10^9^/l, one patient with thrombocytes of 207 × 10^9^/l, familial aHUS, and a *CFB* mutation was included as an exception because of her clear presentation. She was probably diagnosed in an early stage because of her familial background

^c^Reference range 900−1,800 mg/l

^d^Reference range 150−400 mg/l

**Table 3:** Treatment in 45 pediatric atypical hemolytic uremic syndrome (aHUS) patients with mutations in *CFH*, *CFI*, *MCP*, *CFB,* and *C3*, with a homozygous deletion in *CFHR1/3* (*∆CFHR1/3*) and autoantibodies against CFH (αFH), as well as in patients without known genetic anomalies, and the outcomes of their first aHUS episode

Parameter	All patients (no.)	No genetic defect (no.)	Genetic defect (no.)	*CFH* (no.)	*CFI* (no.)	*MCP* (no.)	*CFB* (no.)	*C3* (no.)	*∆CFHR1/3* (no.)	αFH (no.)
Treatment										
Plasma therapy^a^	27 (45)	15 (25)	12 (20)	3 (5)	2 (3)	3 (4)	2 (2)	1 (4)	3 (5)	4 (6)
Dialysis	20 (45)	12 (25)	8 (20)	2 (5)	1 (3)	2 (4)	1 (2)	2 (4)	1 (5)	1 (6)
PT and dialysis	11 (45)	8 (25)	3 (20)	0 (5)	0 (3)	2 (4)	1 (2)	0 (4)	0 (5)	0 (6)
No PT or dialysis	9 (45)	6 (25)	3 (20)	2 (5)	0 (3)	1 (4)	0 (2)	1 (4)	1 (5)	1 (6)
Outcome										
Complete remission	12 (45)	9 (25)	3 (20)	2 (5)	0 (3)	1 (4)	0 (2)	0 (4)	0 (5)	0 (6)
Partial remission	31 (45)	14 (25)	17 (20)	3 (5)	3 (3)	3 (4)	2 (2)	4 (4)	5 (5)	5 (6)
Chronic PT	12 (44)	6 (25)	6 (20)	2 (5)	1 (3)	1 (4)	0 (2)	0 (4)	3 (5)	3 (6)
Chronic dialysis	7 (45)	3 (25)	4 (20)	2 (5)	1 (3)	0 (4)	1 (2)	1 (4)	0 (5)	0 (6)
Death	1 (45)	1 (25)	0 (20)	0 (5)	0 (3)	0 (4)	0 (2)	0 (4)	0 (5)	0 (6)

The numbers of patients for whom data were available are reported in parentheses

*PT* plasma therapy

^a^Plasma infusion and/or exchange

**Table 4:** Long-term patient and renal graft outcomes in 45 pediatric atypical hemolytic uremic syndrome (aHUS) patients with mutations in *CFH*, *CFI*, *MCP*, *CFB,* and *C3*, with a homozygous deletion in *CFHR1/3* (*∆CFHR1/3*) and autoantibodies against CFH (αFH), as well as in patients without known genetic anomalies

Parameter	All patients (no.)	No genetic defect (no.)	Genetic defect (no.)	*CFH* (no.)	*CFI* (no.)	*MCP* (no.)	*CFB* (no.)	*C3* (no.)	*∆CFHR*1/3 (no.)	αFH (no.)
Relapses	21 (43)	9 (23)	12 (20)	3 (5)	1 (3)	2 (4)	1 (2)	3 (4)	3 (5)	3 (6)
No. of relapses	43 (21)	14 (9)	29 (20)	8 (3)	4 (1)	3 (2)	1 (1)	8 (3)	9 (3)	9 (3)
Hypertension	23 (43)	12 (23)	11 (19)	2 (4)	1 (3)	2 (4)	2 (2)	4 (4)	3 (5)	4 (6)
Proteinuria	18 (38)	9 (21)	9 (17)	0 (2)	1 (3)	1 (2)	2 (2)	2 (4)	2 (5)	2 (6)
Renal Tx	7 (44)	3 (24)	4 (20)	2 (5)	1 (3)	1 (4)	0 (2)	1 (4)	0 (5)	0 (6)
No. of Tx	13 (7)	5 (3)	8 (4)	4 (2)	3 (1)	1 (1)	0(0)	3 (1)	0 (0)	0 (0)
No. of relapses in renal graft	10 (13)	4 (5)	6 (8)	5 (4)	2 (3)	0 (1)	0 (0)	2 (3)	0 (0)	0 (0)
Death	4 (45)	2 (25)	2 (20)	0 (5)	0 (3)	1 (4)	0 (2)	0 (4)	1 (5)	1 (6)

The numbers of patients for whom data were available are reported in parentheses

*Tx* transplantation(s)

**Table 5:** Characteristics at disease onset, during treatment, and outcome of four pediatric atypical hemolytic uremic syndrome (aHUS) patients with combined mutations and/or with a homozygous deletion in *CFHR1/3* (*∆CFHR1/3*) and/or autoantibodies against *CFH* (αFH)

Parameter	Patient 1	Patient 2	Patient 3	Patient 4
Combined mutation	*CFI/MCP*	*CFI/C3*	*CFI/∆CFHR1-3/αFH*	*C3/*αFH
Gender	Male	Male	Male	Female
Age at onset (years)	13 10/12	1 2/12	6 6/12	6/12
Hemoglobin (g/dl)	7.2	6.3	9.7	5.5
Platelet count (×10^9^/l)	13	276	120	44
Creatinine (mmol/l)	94	76	294	72
C3 (mg/l)^a^	1000	–	610	670
C4 (mg/l)^b^	185	–	200	–
Plasmatherapy	yes	No	yes	Yes
Dialysis	No	Yes	No	No
Complete remission	No	No	No	No
Partial remission	Yes	Yes	Yes	Yes
Chronic plasmatherapy	No	No	Yes	No
Chronic dialysis	No	Yes	No	No
Death	No	No	No	No
Relapse	No	Yes	No	No
No. of relapses	–	4	–	–
Renal transplant	No	Yes	No	No
No. of renal transplants	–	3	–	–
No. of relapses in grafts	–	2	–	–
Death	No	No	No	No

The numbers of patients for whom data were available are reported in parentheses

^a^Reference range 900−1,800 mg/l

^b^Reference range 150−400 mg/l

### Genetic anomalies

In 20 of the 45 patients (44%) heterozygous mutations in complement regulating genes and/or autoantibodies against CFH were found, all but one with a homozygous deletion in *CFHR1/3* (Table 1). Five patients (overall 11%) carried a mutation in *CFH*, four (overall 9%) in *MCP* and also four in *C3* (overall 9%). Three patients (overall 7%) had a mutation in *CFI* and two (overall 4%) in *CFB*. Six patients (overall 13%) had autoantibodies against CFH. Two of these patients with a genetic anomaly carried a combined mutation (*CFI/MCP* and *CFI/C3*). One patient had a *CFI* mutation and αFH with *∆CFHR1/3,* and in one patient αFH were seen in combination with a *C3* mutation but without *∆CFHR1/3.* Patients with combined mutations are included in both their mutation groups in Tables 2, 3, 4. Characteristics at onset, treatment, and outcome of the four patients with combined genetic anomalies are also summarized separately (Table 5). No mutations in *THBD* were found in our cohort.

### Clinical characteristics

#### Familial vs sporadic

Eleven patients (24%) had familial aHUS, of whom all except two had an associated mutation. We included one family with three sisters with a *CFH* mutation and another family with two cousins with a mutation in *CFB*. Of the other six patients with familial aHUS, only one family member was included in this study because further data were not available. All four patients in this study with a *C3* mutation have familial aHUS. Sporadic aHUS was seen in all four patients with *MCP* mutation and all five patients with αFH and *∆CFHR1/3*.

#### Onset

In ten patients (22%), aHUS onset was before 1 year of age, with the youngest patient being only 1 month old. In only one of these patients was a mutation found (*C3/*αFH), and only two patients had familial aHUS. The majority of aHUS patients (53%) presented between the age of 1 and 7 years, which is also the most common age for children with typical HUS to present with the disease, which suggests that age at onset cannot always help distinguish between typical and aHUS. In 38/45 patients aHUS onset followed a triggering event, such as gastrointestinal symptoms (74%), upper respiratory tract infection (45%), and fever (32%). A combination of triggering events was seen as well; for instance, vomiting and fever were seen in 8/45 patients. Diarrhea was seen in 11 patients, five of whom also had fever. One patient developed aHUS a few days after a hepatitis B vaccination, and one patient was positively tested for a *Bordetella pertussis* infection a few days before aHUS onset. Other triggers were infection with *Haemophilus influenza* (*n* = 1) and a streptococcal infection (*n* = 1).

Hypertension (blood pressure >95th percentile for age) was present at first presentation in 71% of patients. Six patients had involvement of the central nervous system due to malignant hypertension or cerebral infarction during their first aHUS flare. Two of these patients had a mutation (*C3* and *CFB*) and one patient had αFH with *∆CFHR1/3*. These three patients had severe seizures; the other three patients were extremely agitated or had reduced consciousness. One patient had additional visual handicap, and one showed apraxia and aphasia. Pancreas involvement during the first episode was seen in only two patients, both without a genetic defect. In 18 of the 37 patients (49%) for whom data were available, biochemical evaluation showed reduced C3 levels (minimum 210 mg/l, reference range 900–1,800 mg/l). Four of these patients had a mutation (2x*CFB*, 1x*CFI*, 1x*C3*), and four had αFH, of which three also had *∆CFHR1/3.* Low C3 levels were always linked with high levels of the activation product C3d. However, we also found elevated levels of C3d in six patients with normal levels of C3. Of the 25 genetically undefined patients, 11 showed reduced C3 levels, and in seven, elevated levels of C3d were found, showing evidence of alternative pathway activation. Remarkably reduced C4 levels were seen in six of the 31 tested patients (19%), with an extreme lowest level of 30 mg/l (reference range 150–400 mg/l). A systemic disease, such as systemic lupus erythamatosis (SLE) or cryoglobulinemia as cause of these low C4 levels was ruled out because of the clinical and laboratory results combined with medical history. One of these patients had a *CFH* mutation.

### Treatment and outcome of first episode

The majority of patients (60%) were treated with plasma therapy, including plasma infusion (PI) and/or plasma exchange (PE) (Table 2). Plasma therapy was applied in 12 patients with a genetic defect vs 15 patients without a known genetic anomaly. In 11 patients, plasma therapy was combined with dialysis. Nine patients were treated conservatively, three of whom had a genetic defect (1x*C3*, 1x*MCP*, 1xαFH with ∆*CFHR1/3*). In only 3/20 patients (15%) with a genetic defect (2x*CFH*, 1x*MCP*) was complete remission reached after the first episode, compared with 9/24 patients (38%) in the other group. One of these patients (*CFH* mutation) was treated with plasma therapy (four infusions with fresh frozen plasma), one with dialysis (*CFH* mutation), and one patient recovered spontaneously (mutation in *MCP*). All other patients with a genetic anomaly reached partial remission after the first flare. Of the nine patients without plasma therapy or dialysis, only three reached complete remission (1x*MCP*, 2xno genetic anomaly).

Chronic plasma therapy and/or dialysis was applied more often in the group with than in the group without a genetic defect (40% vs 30%). One patient died during the first aHUS episode due to consequences of the disease. This was the youngest patient in the study, only 1 month old. A genetic defect in complement proteins was not found in this patient.

### Long-term outcome

Almost half of the aHUS patients (*n* = 21) had a relapse (Table 3). In the group with a genetic anomaly, 13/20 (65%) of patients relapsed. In the group without known anomalies, relapse occurred in only 8/25 (32%) patients. A total of 43 relapses was seen, of which 29 (67%) occurred in patients with any of the genetic subtypes. A maximum of five relapses was seen in one patient with αFH and *∆CFHR1/3*. Of all patients with a relapse, 38% had familial aHUS. Most relapses occurred after a viral infection, usually of the upper respiratory tract. Time between aHUS onset and the first relapse varied widely, from 1 month after remission to a maximum of 8.5 years. In 9/21 patients, aHUS relapsed within 1 year, five had a genetic defect (1x*CFH*, 1x*MCP*, 1x*C3/CFI*, and 2xαFH with *∆CFHR1/3*). Ten patients with a relapse were initially treated with plasma therapy, eight of whom received chronic plasma therapy. In most patients, plasma therapy was intensified during the relapse.

In 54% of patients, chronic hypertension was seen after the first flare; in 47%, proteinuria sustained. Most of these patients had to be treated for several months with antihypertensive agents. In four patients, neurological involvement was seen later during the course of disease. Two of these patients presented with seizures due to malignant hypertension (*MCP* and *C3*/*CFI*); one patient without a genetic defect had reduced consciousness [computed tomography (CT) scan showed no abnormalities], and one patient had dysphasia and sensibility disorders due to an arteria cerebralis media stenosis (*CFH*). Another patient developed necrotizing pancreatitis with transient diabetes mellitus 10 years after first presentation, for which a partial pancreatectomy was performed. No mutation or deletion was found in this patient.

Seven patients (2x*CFH*, 1x*MCP*, 1x*C3/CFI*, and three without a known genetic defect) received a total number of 13 renal transplants, but in 10/13 grafts, aHUS recurred. Three of these patients had familial aHUS. No recurrence was seen in patient with *MCP* mutation. Time between transplantation and first relapse in renal graft patients varied between 6 days and 4 years. Additionally, one graft was lost due to arteria renalis thrombosis 1 day after transplantation; one graft was lost due to rejection after 4 years. One patient died during the acute phase of aHUS. Three other patients died later during the course of disease. One patient with no genetic defect died 9 years after onset because of massive cerebral bleeding due to malignant hypertension. Another patient with an *MCP* mutation, who had no recurrence of aHUS in the renal transplant, developed pulmonary veno-occlusive disease (PVOD), which led eventually to the finding of an inborn error of cobalamin metabolism, and he died 7.5 years after his first presentation [21]. The last patient died 12 years after aHUS onset but not due to the consequences of aHUS.

## Discussion

In this study, we found that in 45 children diagnosed with aHUS, genetic disorders in complement-regulating genes encoding *CFH*, *CFI*, *MCP*, *CFB*, *C3*, and *THBD*, as well as the presence of αFH with or without a homozygous deletion in *CFHR1/3*, are linked with clinical presentation, treatment, and outcome. In almost 50% of this pediatric aHUS cohort, a genetic defect in complement-regulating genes was found. This finding is compatible with the previous French pediatric report of Sellier-Leclerc et al. and the Italian adult/pediatric registry reported by Noris et al. [3, 6]. Most mutations (overall 13%) were found in *CFH*, which corresponds to the literature [3, 5, 6, 9]. The number of mutations in *MCP* and *C3* were equal (overall 9%). Fewer mutations (overall 7%) were found in *CFI*, just as seen in the pediatric French cohort. Mutations in *CFB* are relatively rare [6, 9] and according to the literature occur in only 1–2% of patients with aHUS [2]. We identified a genetic aberration in *CFB* in 4.4% of our patients, but it must be mentioned that these two patients were related. Delvaeye et al. report that about 5% of patients with aHUS have mutations that impair thrombomodulin function [6, 19]. Remarkably, in our cohort, no aHUS-associated thrombomodulin mutations were found.

Dragon-Durey et al. were the first to describe the association between αFH and aHUS [15], and more recently an association between the presence of αFH and a homozygous polymorphic deletion in *CFHR1/3* was described [17]. In our population, 13% of all patients had αFH, and 83% of these patients (overall 11%) had an additional *∆CFHR1/3*, which corresponds well to previous published data [15–18].

One of the most important observations in this cohort is that 74% of aHUS patients showed gastrointestinal symptoms, of which 25% presented with diarrhea. This clearly shows that postdiarrheal onset does not exclude the possibility of aHUS. This makes differentiating between typical HUS and aHUS for the general pediatrician sometimes even more difficult and may even result in a delay of adequate treatment in these patients. Also, relapses seem to occur most often after a triggering event, usually an upper airway infection or gastroenteritis. Remarkably, in one patient, aHUS relapsed twice after a cytomegalovirus infection for which daily PE and ganciclovir were initiated [22]. In one other patient, aHUS onset followed a hepatitis B vaccination a few days earlier. The triggering role of vaccination in this patient was confirmed by a relapse shortly after combined anti-diphtheria-pertussis-tetanus-polio vaccination.

In only 18 of the 37 tested patients (49%) were C3 plasma levels reduced, which indicates, as shown by others, that normal C3 levels in aHUS patients do not reflect complement activation on the endothelial cell surfaces. Normal C3 levels also do not rule out the existence of mutations in genes encoding complement-regulating proteins. Breakdown products of C3 activation, such as C3d, as well as soluble complement complex C5b-9 (SC5b-9), might be a better serum marker for complement activation in aHUS patients.

Although at this time results of the use of eculizumab in patients with aHUS look very promising [23, 24] and might replace plasma therapy in the near future, at present, plasma therapy is considered the gold standard treatment in patients with aHUS and should be started within 24 h of diagnosis [25]. However, only 60% of our population received plasma therapy (with or without dialysis). This is due to the fact that in our study, patients were included at the time their DNA was sent to the laboratory between 2001 and 2010, a time when knowledge about aHUS treatment was growing. However, those patients’ first or even second HUS episode was sometimes long before that period, when no clear therapy schedules were available. Of the patients treated without plasma therapy, 44% were diagnosed and already treated before 2000. Only in recent years have new treatment guidelines for aHUS been published [25]. Since the latest guideline in 2008, every patient in this study was treated with plasma therapy.

In our cohort, seven patients received a total number of 13 kidney transplants. In five of these seven patients, transplantation was performed before the existence of genetic complement mutations was known and before plasmapheresis as treatment of aHUS was well accepted. Four of these patients had a genetic defect (1x*MCP*, 2x*CFH*, and 1x*CFI/C3*). All except one (*MCP*) graft failed (93%), again demonstrating that kidney transplantation is compromised by the risk of recurrence [2, 3, 5, 6, 26, 27]. Renal transplantation in patients with aHUS should not be performed without DNA analysis for all involved complement genes due to the fact that this leads to a better defined risk profile for relapses.

Currently, aHUS patients having a *CFH* or *CFI* mutation who will have a single kidney transplantation are advised to combine this with prophylactic plasmapheresis and life-long PE [28], or else these patients are advised to have a combined liver–kidney transplantation with preoperative PE, intraoperative PI, and posttransplant anticoagulation [27]. In the near future, one might expect that lifelong prophylactic treatment with complement inhibitors will replace the use of plasma and /or the combination of plasma with liver and/or renal transplantation in aHUS patients with *CFH* or *CFI* mutations [23, 24].

In conclusion, in almost 50% of pediatric aHUS patients, a genetic abnormality was found. In 87% of patients, aHUS onset followed a triggering event; 25% of cases presented with diarrhea, which makes differentiating between typical and aHUS difficult. Half of the patients (21/43) had a relapse (67% of them with a genetic anomaly), and all except one renal graft failed, so renal transplantation should not be performed before adequate DNA analysis of the involved complement genes. In the near future, next-generation sequencing methods, such as whole exome or genome sequencing, will more easily facilitate DNA mutation analysis, and new genes will probably be identified that might be associated with aHUS as well. With the use of complement inhibitors in the ongoing trials in aHUS patients, a new era in the treatment of patients with aHUS has begun. It has yet to be demonstrated whether this treatment will improve the overall results in aHUS patients and hopefully reduce the requirement for dialysis and renal transplantations.

## References

[1] Ruggenenti P, Noris M, Remuzzi G (2001). Thrombotic microangiopathy, hemolytic uremic syndrome, and thrombotic thrombocytopenic purpura. Kidney Int.

[2] Noris M, Remuzzi G (2009). Atypical hemolytic-uremic syndrome. N Engl J Med.

[3] Sellier-Leclerc AL, Fremeaux-Bacchi V, Dragon-Durey MA, Macher MA, Niaudet P, Guest G, Boudailliez B, Bouissou F, Deschenes G, Gie S, Tsimaratos M, Fischbach M, Morin D, Nivet H, Alberti C, Loirat C (2007). Differential impact of complement mutations on clinical characteristics in atypical hemolytic uremic syndrome. J Am Soc Nephrol.

[4] Sharma AP, Greenberg CR, Prasad AN, Prasad C (2007). Hemolytic uremic syndrome (HUS) secondary to cobalamin C (cblC) disorder. Pediatr Nephrol.

[5] Caprioli J, Noris M, Brioschi S, Pianetti G, Castelletti F, Bettinaglio P, Mele C, Bresin E, Cassis L, Gamba S, Porrati F, Bucchioni S, Monteferrante G, Fang CJ, Liszewski MK, Kavanagh D, Atkinson JP, Remuzzi G (2006). Genetics of HUS: the impact of MCP, CFH, and IF mutations on clinical presentation, response to treatment, and outcome. Blood.

[6] Noris M, Caprioli J, Bresin E, Mossali C, Pianetti G, Gamba S, Daina E, Fenili C, Castelletti F, Sorosina A, Piras R, Donadelli R, Maranta R, van der Meer I, Conway EM, Zipfel PF, Goodship TH, Remuzzi G (2010). Relative role of genetic complement abnormalities in sporadic and familial aHUS and their impact on clinical phenotype. Clin J Am Soc Nephrol.

[7] Richards A, Kemp EJ, Liszewski MK, Goodship JA, Lampe AK, Decorte R, Müslümanoğlu MH, Kavukcu S, Filler G, Pirson Y, Wen LS, Atkinson JP, Goodship TH (2003). Mutations in human complement regulator, membrane cofactor protein (CD46), predispose to development of familial hemolytic uremic syndrome. Proc Natl Acad Sci USA.

[8] Fremeaux-Bacchi V, Moulton EA, Kavanagh D, Dragon-Durey MA, Blouin J, Caudy A, Arzouk N, Cleper R, Francois M, Guest G, Pourrat J, Seligman R, Fridman WH, Loirat C, Atkinson JP (2006). Genetic and functional analyses of membrane cofactor protein (CD46) mutations in atypical hemolytic uremic syndrome. J Am Soc Nephrol.

[9] Westra D, Volokhina E, van der Heijden E, Vos A, Huigen M, Jansen J, van Kaauwen E, van der Velden T, van de Kar N, van den Heuvel L (2010). Genetic disorders in complement (regulating) genes in patients with atypical haemolytic uraemic syndrome (aHUS). Nephrol Dial Transplant.

[10] Fremeaux-Bacchi V, Dragon-Durey MA, Blouin J, Vigneau C, Kuypers D, Boudailliez B, Loirat C, Rondeau E, Fridman WH (2004). Complement factor I: a susceptibility gene for atypical haemolytic uraemic syndrome. J Med Genet.

[11] Kavanagh D, Kemp EJ, Mayland E, Winney RJ, Duffield JS, Warwick G, Richards A, Ward R, Goodship JA, Goodship TH (2005). Mutations in complement factor I predispose to development of atypical hemolytic uremic syndrome. J Am Soc Nephrol.

[12] Goicoechea de Jorge E, Harris CL, Esparza-Gordillo J, Carreras L, Arranz EA, Garrido CA, López-Trascasa M, Sánchez-Corral P, Morgan BP, Rodríguez de Córdoba S (2007). Gain-of-function mutations in complement factor B are associated with atypical hemolytic uremic syndrome. Proc Natl Acad Sci USA.

[13] Caprioli J, Remuzzi G (2007). Complement hyperactivation may cause atypical haemolytic uraemic syndrome–gain-of-function mutations in factor B. Nephrol Dial Transplant.

[14] Frémeaux-Bacchi V, Miller EC, Liszewski MK, Strain L, Blouin J, Brown AL, Moghal N, Kaplan BS, Weiss RA, Lhotta K, Kapur G, Mattoo T, Nivet H, Wong W, Gie S, Hurault de Ligny B, Fischbach M, Gupta R, Hauhart R, Meunier V, Loirat C, Dragon-Durey MA, Fridman WH, Janssen BJ, Goodship TH, Atkinson JP (2008). Mutations in complement C3 predispose to development of atypical hemolytic uremic syndrome. Blood.

[15] Dragon-Durey MA, Loirat C, Cloarec S, Macher MA, Blouin J, Nivet H, Weiss L, Fridman WH, Frémeaux-Bacchi V (2005). Anti-Factor H autoantibodies associated with atypical hemolytic uremic syndrome. J Am Soc Nephrol.

[16] Zipfel PF, Edey M, Heinen S, Józsi M, Richter H, Misselwitz J, Hoppe B, Routledge D, Strain L, Hughes AE, Goodship JA, Licht C, Goodship TH, Skerka C (2007). Deletion of complement factor H-related genes CFHR1 and CFHR3 is associated with atypical hemolytic uremic syndrome. PLoS Genet.

[17] Józsi M, Licht C, Strobel S, Zipfel SL, Richter H, Heinen S, Zipfel PF, Skerka C (2008). Factor H autoantibodies in atypical hemolytic uremic syndrome correlate with CFHR1/CFHR3 deficiency. Blood.

[18] Moore I, Strain L, Pappworth I, Kavanagh D, Barlow PN, Herbert AP, Schmidt CQ, Staniforth SJ, Holmes LV, Ward R, Morgan L, Goodship TH, Marchbank KJ (2010). Association of factor H autoantibodies with deletions of CFHR1, CFHR3, CFHR4, and with mutations in CFH, CFI, CD46, and C3 in patients with atypical hemolytic uremic syndrome. Blood.

[19] Delvaeye M, Noris M, De Vriese A, Esmon CT, Esmon NL, Ferrell G, Del-Favero J, Plaisance S, Claes B, Lambrechts D, Zoja C, Remuzzi G, Conway EM (2009). Thrombomodulin mutations in atypical hemolytic-uremic syndrome. N Engl J Med.

[20] Józsi M, Strobel S, Dahse HM, Liu WS, Hoyer PF, Oppermann M, Skerka C, Zipfel PF (2007). Anti factor H autoantibodies block C-terminal recognition function of factor H in hemolytic uremic syndrome. Blood.

[21] Bouts AH, Roofthooft MT, Salomons GS, Davin JC (2010). CD46-associated atypical hemolytic uremic syndrome with uncommon course caused by cblC deficiency. Pediatr Nephrol.

[22] Olie KH, Goodship TH, Verlaak R, Florquin S, Groothoff JW, Strain L, Weening JJ, Davin JC (2005). Posttransplantation cytomegalovirus-induced recurrence of atypical hemolytic uremic syndrome associated with a factor H mutation: successful treatment with intensive plasma exchanges and ganciclovir. Am J Kidney Dis.

[23] Davin JC, Gracchi V, Bouts A, Groothoff J, Strain L, Goodship T (2010). Maintenance of kidney function following treatment with eculizumab and discontinuation of plasma exchange after a third kidney transplant for atypical hemolytic uremic syndrome associated with a CFH mutation. Am J Kidney Dis.

[24] Weitz M, Amon O, Bassler D, Koenigsrainer A, Nadalin S (2011). Prophylactic eculizumab prior to kidney transplantation for atypical hemolytic uremic syndrome. Pediatr Nephrol.

[25] Ariceta G, Besbas N, Johnson S, Karpman D, Landau D, Licht C, Loirat C, Pecoraro C, Taylor CM, Van de Kar N, Vandewalle J, Zimmerhackl LB (2009). Guideline for the investigation and initial therapy of diarrhea-negative hemolytic uremic syndrome. Pediatr Nephrol.

[26] Bresin E, Daina E, Noris M, Castelletti F, Stefanov R, Hill P, Goodship TH, Remuzzi G (2006). Outcome of renal transplantation in patients with non-Shiga toxin-associated hemolytic uremic syndrome: prognostic significance of genetic background. Clin J Am Soc Nephrol.

[27] Noris M, Remuzzi G (2010). Thrombotic microangiopathy after kidney transplantation. Am J Transplant.

[28] Davin JC, Strain L, Goodship TH (2008). Plasma therapy in atypical haemolytic uremic syndrome: lessons from a family with a factor H mutation. Pediatr Nephrol.

[29] Roumenina LT, Frimat M, Miller EC, Provot F, Dragon-Durey MA, Bordereau P, Bigot S, Hue C, Satchell SC, Mathieson PW, Mousson C, Noel C, Santes-Fridman C, Halbwachs-Mecarelli L, Atkinson JP, Liomet A, Fremeaux-Bacchi V (2012) A prevalent C3 mutation in a HUS patients causes a direct C3 convertase gain-of-function. Blood. doi:10.1182/blood-2011-10-383281 10.1182/blood-2011-10-383281 PMC3359738 22246034

